# Supplementary material for: Preprint articles as a tool for teaching data analysis and scientific communication
Source: PLoS One. 2021 Dec 21;16(12):e0261622. doi: 10.1371/journal.pone.0261622 (PMC8691640; doi:10.1371/journal.pone.0261622)
Supplement: S1 File — (PDF) [file pone.0261622.s001.pdf]

Worksheet Part 1: Paper Analysis

Please upload your work to the online course management system.

Use this template to find the most important information from the paper.
Be careful about plagiarism, and be sure to <u>rephrase the information</u> in your own words

1. Introduction section

Briefly state the **<u>main problem(s)</u>** being addressed in the paper. (Often the paper will denote the main problem using the phrases “The problem…”, “The challenge…”, “Our current limitation…”, Currently, we cannot…”)

What is the **<u>importance to the field</u>**? Often the paper will indicate what the current research will enable or allow us to do in the future.

What **<u>background information</u>** is necessary to know in order to understand the main problem being addressed in the paper? What research has previously been performed in this area that informs the authors as they begin their study (often this information can be provided by review articles that are related to your article).

<u>Restate the specific problem</u> or hypothesis being covered in the paper. The paper may use the phrases “Our goal was to….”, “we sought to…”, “we aimed to…”

2. Results: Complete one row in the table for each experiment. Note that for figures with multiple panels (Figure 2a, 2b, etc) you may want to use a separate row to describe each, especially if different techniques were used. Add rows as needed (click the table and select Layout at the top of Word and then Insert Below)

Figure number	Purpose of experiment	Technique used or variables manipulated	What results are obtained from the experiment?	Critique the experiment-Does everything look good or are there controls that should be added, statistical tests that should be run, data that is not interpreted properly?

3. Discussion:

Summary of the conclusions. In 1-2 sentences, what did the authors find in the paper? Often they will include this summary at the start of the discussion section.

What was the **importance** of the research? Do the authors of this paper tell you how the research compares to previous results? Are the results consistent with what has been seen previously or are they novel or unexpected?

Do they authors propose a new mechanism, model, or application? Do they discuss what the **future problems or directions** in the research area should be?
