# Supplementary material for: Preprint articles as a tool for teaching data analysis and scientific communication
Source: PLoS One. 2021 Dec 21;16(12):e0261622. doi: 10.1371/journal.pone.0261622 (PMC8691640; doi:10.1371/journal.pone.0261622)
Supplement: S2 File — (PDF) [file pone.0261622.s002.pdf]

Worksheet Part 2: Comparison of the Preprint and Final Versions of the Article

Compare the preprint version of the article and the final version. Analyze the introduction, results, and discussion sections only (not the materials and methods, references, etc). It will be easiest to make the comparison 3-4 sentences at a time. Note all important changes in the table below. How can you tell if a change is important?

What's not important:

- Changes in grammar, sentence structure, abbreviations, capitalization, or wording that do not affect the meaning
- Changes in the numbering of figure (ie what used to be Figure 2 is now Figure 3)
- Changes to the references (may be important, but we won't deal with them now)
- Movement of text or figures to now be part of the supplemental information

Other changes are important, and you should note the following about them in the table below (add additional rows to the table as needed):

What type of change was it?

- An addition
- A change in information
- A deletion

What portion of the paper did it affect?

- Background information in the introduction
- The data/results from experiments that this group performed
- Statistical tests or validation
- How the data is interpreted/what the data is stated to mean
- Conclusions that are drawn about what the data means or why it is important
- **If you find at least 5 significant examples of one type of change (ie 5 changes to the background information, 5 changes to the data or results), you can stop finding additional examples for that type of change. Your work does not need to be exhaustive.**

If you are not sure whether a particular type of change is important or not, <u>please ask</u>!!

Section of Paper (Intro, Results, Discussion)	Paragraph of that section	What type of change was it?	What portion of the paper did it affect?	Specifics of change	Why do you speculate that this change was made?

