# Supplementary material for: Preprint articles as a tool for teaching data analysis and scientific communication
Source: PLoS One. 2021 Dec 21;16(12):e0261622. doi: 10.1371/journal.pone.0261622 (PMC8691640; doi:10.1371/journal.pone.0261622)
Supplement: S3 File — (PDF) [file pone.0261622.s003.pdf]

Worksheet Part 3: Overall Impressions of Scientific Review

For the preprint, what was the date on which it was posted?

For the final paper, what was the date of submission?

For the final paper, what was the date of acceptance?

For the final paper, on what date was it published?

Please use 3-5 sentences to answer each question below

1. What surprised you most when you compared the preprint and final versions of the paper?

2. Is it easy or hard to distinguish which additions were made because the authors had more time to acquire more data and additions that were made due to requests from the peer reviewers? How do you think you can you tell the two apart?

3. What, if anything, did you learn about the scientific publishing process from this activity?

4. What, if anything, did you learn about presenting and interpreting research data from this activity?

5. Many preprint articles present data that are important advances into SARS-coV2 research, and it can be important to get the information out in a timely manner. However, it is also crucial that the research be reliable and accurate. Do you think that preprint servers are a good way to get results out in a hurry?

6. Go to the preprint server and look at the usage metrics for your article. How many times were the HTML and PDF versions of the preprint downloaded? How many times was it picked up by news outlets, blogged about, or tweeted? Do you feel that the level of attention that this non-peer reviewed article received was appropriate?

7. Do you think that this paper and the publishing process that it went through achieved an appropriate balance between getting research results out quickly in a pandemic situation versus the need for high-quality, complete, and reproducible research? Should we maintain this process, or are there changes that should be implemented?
