# Supplementary material for: Preprint articles as a tool for teaching data analysis and scientific communication
Source: PLoS One. 2021 Dec 21;16(12):e0261622. doi: 10.1371/journal.pone.0261622 (PMC8691640; doi:10.1371/journal.pone.0261622)
Supplement: S4 File — (PDF) [file pone.0261622.s004.pdf]

Worksheet Part 1: Paper Analysis

Please complete by **Sept 9th** and upload your work to Moodle

Use this template to find the most important information from the paper.
Be careful about plagiarism, and be sure to rephrase the information in your own words

1. Introduction section

Briefly state the **main problem(s)** being addressed in the paper. (Often the paper will denote the main problem using the phrases “The problem…”, “The challenge…”, “Our current limitation…”, Currently, we cannot…”)

We are not able to assemble and maintain the full-length clones of large RNA virus genomes in *E. coli.*

What is the **importance to the field**? Often the paper will indicate what the current research will enable or allow us to do in the future.

It will allow us to learn more about RNA virus variants (such as SARS-CoV-2) during outbreaks.

What **background information** is necessary to know in order to understand the main problem being addressed in the paper? What research has previously been performed in this area that informs the authors as they begin their study (often this information can be provided by review articles that are related to your article).

In order for an RNA virus to be located in a eukaryotic cell, it must be transcribed from the cloned DNA template. For viruses in the *Coronaviridae* family with large genome sizes, the clones are often damaged. Cloning in other approaches such as vaccinia virus, subgenomic DNA fragments with *in*-vitro ligation, and bacterial artificial chromosomes (BACs) have been successful, however, they all still leave the coronaviruses hard to manage.

Restate the specific problem or hypothesis being covered in the paper. The paper may use the phrases “Our goal was to….”, “we sought to…”, “we aimed to…”

They aimed to find out if yeast *S. cerevisiae* was able to assemble and maintain different RNA viruses so these viruses could be studied further.

2. Results: Complete one row in the table for each experiment. Note that for figures with multiple panels (Figure 2a, 2b, etc) you may want to use a separate row to describe each, especially if different techniques were used. Add rows as needed (click the table and select Layout at the top of Word and then Insert Below)

Figure number	Purpose of experiment	Technique used or variables manipulated	What results are obtained from the experiment?	Critique the experiment-Does everything look good or are there controls that should be added, statistical tests that should be run, data that is not interpreted properly?
1a-c	Testing the yeast-based platform for recovery of RNA viruses	They used the murine hepatitis virus with the gene for a green fluorescent protein (MHV-GFP). They PCR-amplified 9 overlapping DNA fragments and used TAR cloning to deliver the DNA into the yeast. The fragments were then recombined to create the YAC-cloned full-length cDNA. Next, the DNA was linearized *in-vitro.* Finally, the virus was rescued using electroporation of the cells expressing GFP. Two clones were taken and used to infect murine cells. These cells were then cultured and analyzed for GFP expression.	The clones assembled in the yeast showed that they infected the murine cells with the virus. The yeast-based platform maintained the RNA virus well.	Enough controls and tests were used.
1d	Testing the replication kinetics of the recovered viruses	They looked at cell cultures over time using plaque assays from a parental MHV-GFP and the two clones.	The cells infected by the clones and the cells infected by the parental MHV-GFP follow very similar timelines of plaque formation.	Everything looks good. The experiments were all independent and repeated twice.
2a-c	Testing if the platform can be used with other coronaviruses and rapid mutagenesis	They used a BAC clone of MERS-CoV, PCR-amplified 8 DNA overlapping fragments, introduced GFP, rescued the virus from cloned DNA, and looked for green fluorescence.	VeroB4 cells fluoresced green, showing that coronavirus genomes can be modified using this platform. The replication kinetics of the MERS-CoV and	Everything looks good. Controls were used and the experiments were all independent and repeated twice.

			MERS-CoV-GFP were slightly lower than the MERS-CoV-EMC (lab adapted isolate).	
Extended data Figure 1d-h	Testing if the platform can be used with viruses in other families	The same strategy was used to prepare clones of other coronaviruses as well as viruses in the *Flaviviridae* and *Paramyxoviridae* families.	They were able to clone diverse RNA viruses from many different samples using this method.	For some of the viruses they used, not all of the clones contained a properly assembled genome (e.g. ZIKA only had 3/15 clones with the proper viral genome).
3a-d	Using the reverse genetics platform to analyze SARS-CoV-2	They split SARS-CoV-2 into 12 DNA fragments and added GFP to fragment 11. Using synthetic DNA constructs and viral RNA from a patient, they were able to make 6 clones (3 with GFP). They followed the same procedure as before to analyze the clones.	Green fluorescent cells and plaque were detected.	Enough controls and tests were used.

3. Discussion:

Summary of the conclusions. In 1-2 sentences, what did the authors find in the paper? Often they will include this summary at the start of the discussion section.

Their method of homologous recombination in yeast can provide full-length cDNAs for large RNA viruses that are hard to generate in *E. coli.* Using the TAR cloning system allows the genomes to be split into at least 14 fragments and be reassembled correctly.

What was the **importance** of the research? Do the authors of this paper tell you how the research compares to previous results? Are the results consistent with what has been seen previously or are they novel or unexpected?

Their synthetic genomics approach is fast at generating SARS-CoV-2 and other RNA viruses. Clinical samples are not needed unlike other approaches. This is a new approach.

Do they authors propose a new mechanism, model, or application? Do they discuss what the **future problems or directions** in the research area should be?

SARS-CoV-2 is likely to evolve in humans with sequence variations and phenotypic changes. Using their platform, further research can be done on SARS-CoV-2 without delays so we can keep up with it as it evolves.
