# Supplementary material for: Preprint articles as a tool for teaching data analysis and scientific communication
Source: PLoS One. 2021 Dec 21;16(12):e0261622. doi: 10.1371/journal.pone.0261622 (PMC8691640; doi:10.1371/journal.pone.0261622)
Supplement: S5 File — (PDF) [file pone.0261622.s005.pdf]

Worksheet Part 2: Comparison of the Preprint and Final Versions of the Article

Compare the preprint version of the article and the final version. Analyze the introduction, results, and discussion sections only (not the materials and methods, references, etc). It will be easiest to make the comparison 3-4 sentences at a time. Note all important changes in the table below. How can you tell if a change is important?

What's not important:

- Changes in grammar, sentence structure, abbreviations, capitalization, or wording that do not affect the meaning
- Changes in the numbering of figure (ie what used to be Figure 2 is now Figure 3)
- Changes to the references (may be important, but we won't deal with them now)
- Movement of text or figures to now be part of the supplemental information

Other changes are important, and you should note the following about them in the table below (add additional rows to the table as needed):

What type of change was it?

- An addition
- A change in information
- A deletion

What portion of the paper did it affect?

- Background information in the introduction
- The data/results from experiments that this group performed
- Statistical tests or validation
- How the data is interpreted/what the data is stated to mean
- Conclusions that are drawn about what the data means or why it is important
- **If you find at least 5 significant examples of one type of change (ie 5 changes to the background information, 5 changes to the data or results), you can stop finding additional examples for that type of change. Your work does not need to be exhaustive.**

If you are not sure whether a particular type of change is important or not, <u>please ask</u>!!

**Section of Paper (Intro, Results, Discussion)**	**Paragraph of that section**	**What type of change was it?**	**What portion of the paper did it affect?**	**Specifics of change**	**Why do you speculate that this change was made?**
Intro	Third paragraph	Deletion	Background information	They removed the sentences discussing how the entire genome of DNA viruses are able to be transfected into eukaryotic cells whereas RNA viruses must be transcribed first from a cloned DNA template.	This information was not essential to understand the difference between DNA viruses and RNA viruses.
Results	Fifth paragraph	Change in information	Conclusions about the method	In the preprint, they said the method had full functionality. Now, they admitted that some of the DNA clones and viruses contained an incorrect sequence, but they gave some reasons why that happened.	In the preprint, they only discussed the errors later on. Giving false information about full functionality is definitely not good in published papers.
Discussion	First paragraph	Change in information	Discussion on the capabilities of TAR cloning	The minimum number of overlapping DNA fragments using TAR cloning was 14 but now is 19.	From the time in between the preprint and publication, the researchers might have performed more experiments using TAR cloning.
Methods	The whole section	Addition of a methods section	Added extra information to better explain the experiment and the results	They included their protocol for each step in the experiment. They discussed the specific cells,	Their method of synthesizing genomes in yeast was what they were trying to inform

				volumes, concentrations, materials, times, and reagents they used.	other scientists about. It only makes sense to include the protocol in the paper so people know how to use the method correctly.
Results	Figure 1c	Addition	Statistical test	They conducted a two-sided unpaired t-test for the difference in kinetics between the clones and parental MHV-GFP. All p-values were greater than .o5 so the differences were not significant.	Even though the graphs of the clones versus the parental looked the same, a statistical test should be done to confirm that conclusion so there are no discrepancies.
Results	Figure 3c	Addition	Statistical tests/validation and data from the results.	Graphs of the kinetics of the SARS-CoV-2 and SARS-CoV-2-GFP clones and isolates were included as well as tests of significance.	The researchers had more time to conduct further tests to prove their method works. However, the significance tests for the GFP showed that the clones did not have the same kinetics as the isolate.
Results	Extended Data Figures	Addition	Added more data and statistical tests to the results, giving more proof that the method works	The extended data figures were included to go along with the explanations from the preprint. The new figures also showed new tests that were preformed including	Figure descriptions should always have figures to go with them. Also, they had more time to perform different tests.

				comparisons between SARS-CoV-2 clones with the same 5’ terminus, serum neutralization assays for the synthetic SARS-CoV-2, immunofluorescence assay showing viral protein synthesis, fluorescence assays for SARS-CoV-GFP with Remdesivir and DMSO, and titration for SARS-CoV-GFP.	
