# Supplementary material for: Preprint articles as a tool for teaching data analysis and scientific communication
Source: PLoS One. 2021 Dec 21;16(12):e0261622. doi: 10.1371/journal.pone.0261622 (PMC8691640; doi:10.1371/journal.pone.0261622)
Supplement: Attachment — Submitted filename: Response to Reviewers.pdf [file pone.0261622.s006.pdf]

Loyola University Maryland
— 1852 —
Department of Biology

August 22, 2021

Dear Dr. Soh:

Thank you for your assistance in seeing our manuscript through the peer review process. We appreciate the careful review by the reviewers, their overall strongly positive assessment of our work, and their view that only minor corrections to our manuscript are needed. We have addressed all the reviewers' requests and include a point-by-point response below.

1. Reviewer #2: I would consider making it less extensive in order that readers can locate themselves in the strategy used and the qualitative form that you have used to evaluate the effectiveness of this strategy in Higher Education Teaching.

   While we agree with reviewer 2's statement that it is necessary for readers to be able to see how they could use or implement this pedagogical strategy, we believe that shortening the manuscript and making it less extensive would hinder that process rather than enabling it. Because our pedagogical technique can be implemented in different classes and contexts, we felt that it is necessary to include sufficient details about each of the different instances of the technique being used that a diverse range of faculty from different institutional types can best use this teaching strategy.

2. Reviewer #3: Could you describe the methodological design you used?

   We appreciate the reviewers' request here and have included a better explanation that we believe improves the revised manuscript. Specifically, we described how we designed our study methods correspond to three steps of the cognitive apprenticeship approach (lines 113-118 in the revised manuscript with tracked changes), the learning model on which our pedagogical approach is based.

3. Reviewer #3: Did any students refuse to participate, or did they not complete the entire assessment?

   No students refused to participate, but this exercise was one of two options that students could choose from in one of the participating courses; we have clarified in the manuscript that 6 of the 24 enrolled students chose to participate in this study (lines 98-99 in the revised manuscript with tracked changes). Two students did not complete the entire assignment, and we have now clarified that student work was not included unless all three parts were completed (lines 101-102 in the revised manuscript with tracked changes).

4. Reviewer #3: Regarding the qualitative results, precisely the verbatims, could you provide information about the participant, at least age and gender?

   We appreciate the reviewer's interest in characteristics of our study participants. We describe the gender and class year of all our participants in the methods section. To respond more precisely to this request while still protecting our students' privacy, we have included the students' gender and class year for each of the significant direct quotes, and this information can now be found on lines 257, 281, 323, 391, 429, 480 of the revised manuscript with tracked changes.

4501 North Charles Street, Baltimore, Maryland 21210
Phone 410-617-2000 · www.loyola.edu

5. Reviewer 3: A minor language revision is necessary to improve the manuscript understandable. Below are some grammatical errors to be corrected:

Introduction
Line 25 science to scientific

This correction has been made.

Line 36 step to steps

We believe that this is grammatically correct as written: “publication on a preprint server is increasingly becoming the first step of publishing a research article”.

Line 37 peer reviewed to peer-reviewed

This correction has been made.

Line 62 “Central to this process are the” to “Central to this process is the”

We believe that this is correct as written; the subject of the sentence is “the principles” and therefore the verb should be plural “are”.

Line 68 critique by having students review articles themselves to critiques by having students review articles themselves

This correction has been made.

Line 72 difference between their assessment and the peer reviewer’s. to differences between their assessment and the peer reviewers.

This correction has been made.

Discussion
Line 477 judgement to judgment

This correction has been made.

Thank you again for your help shepherding our manuscript. We hope that these changes have fully addressed the reviewers’ concerns, but if not, we would be happy to make additional revisions as necessary.

Sincerely,

Lisa Scheifele
Associate Professor and Chair
